# Segmentation of Juxtapleural Pulmonary Nodules Using a Robust Surface Estimate

**DOI:** 10.1155/2011/632195

**Published:** 2011-06-26

**Authors:** Artit C. Jirapatnakul, Yury D. Mulman, Anthony P. Reeves, David F. Yankelevitz, Claudia I. Henschke

**Affiliations:** ^1^School of Electrical and Computer Engineering, Cornell University, Ithaca, NY 14853, USA; ^2^Department of Radiology, Mount Sinai School of Medicine, 1 Gustave L. Levy Place, New York, NY 10029, USA

## Abstract

An algorithm was developed to segment solid pulmonary nodules attached to the chest wall in computed tomography scans. The pleural surface was estimated and used to segment the nodule from the chest wall. To estimate the surface, a robust approach was used to identify points that lie on the pleural surface but not on the nodule. A 3D surface was estimated from the identified surface points. The segmentation performance of the algorithm was evaluated on a database of 150 solid juxtapleural pulmonary nodules. Segmented images were rated on a scale of 1 to 4 based on visual inspection, with 3 and 4 considered acceptable. This algorithm offers a large improvement in the success rate of juxtapleural nodule segmentation, successfully segmenting 98.0% of nodules compared to 81.3% for a previously published plane-fitting algorithm, which will provide for the development of more robust automated nodule measurement methods.

## 1. Introduction

One of the most reliable indicators of the malignancy of a pulmonary nodule is its growth rate [1, 2]. To accurately measure the growth rate of a nodule, automated methods need to repeatably and robustly measure the volume of a nodule on several scans. Juxtapleural pulmonary nodules are attached to the chest wall and pleural surface; these nodules present a challenge to many automated measurement algorithms due to the need to decide on a boundary between the nodule and chest wall without the presence of any difference in intensity between the two structures. In contrast, isolated nodules, which do not abut any other structures such as airways or blood vessels, are substantially easier to segment. We developed an automated method to segment juxtapleural nodules using robust surface-fitting techniques.

**Figure 1:**
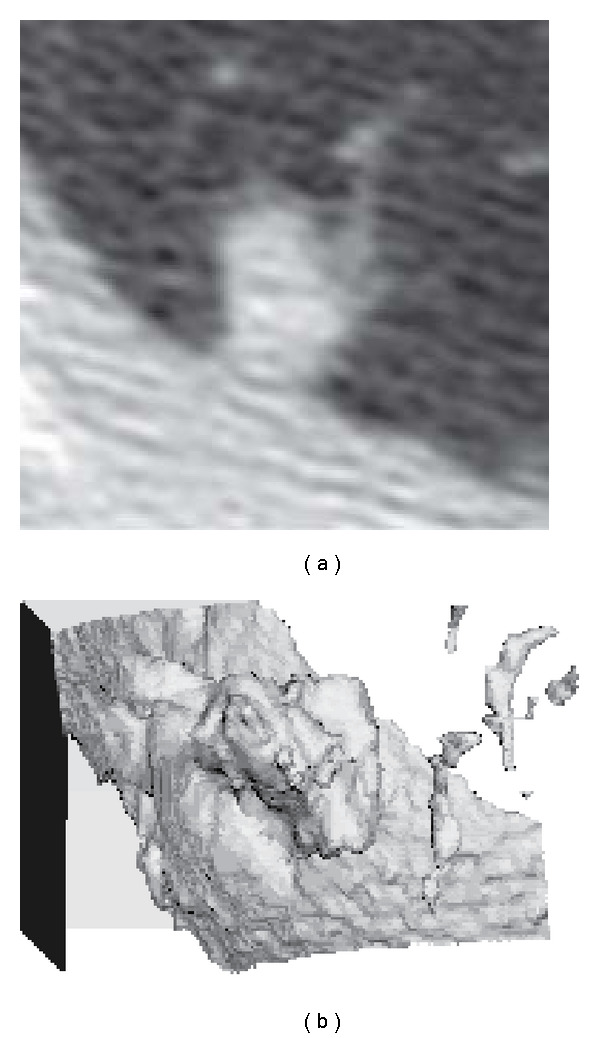
Example of a juxtapleural nodule, with (a) one central slice from CT scan and (b) 3D visualization of region of interest.

 The problem of isolated nodule segmentation has been well studied; isolated nodules can often be segmented via intensity and shape-based methods, as in one method proposed by Zhao et al. [3]. Juxtapleural nodules are more difficult to segment accurately than isolated nodules due to the challenge in determining the location of the invisible boundary between the nodule and the lung wall. An example of a juxtapleural nodule is shown in Figure 1. Note that the nodule is the same intensity as the lung wall, rendering intensity-based segmentation methods ineffective with juxtapleural nodules—instead, a decision for locating the nodule boundary can only be based on properties of adjacent sections of the pleural surface. Previous works in this area have segmented juxtapleural nodules using morphological filtering or various surface-fitting algorithms. Kostis et al. identified the thoracic wall and segmented nodules using morphological filtering with ellipsoid kernels [4]. Reeves et al. modeled the pleural surface with a plane, using an iterative procedure to find the optimal parameters for a plane that separates the nodule from the surface [5]. Way et al. used an active contour approach to segment 23 isolated and attached nodules from the LIDC database [6]. Another approach by Okada et al. [7] used robust anisotropic Gaussian fitting followed by a morphological opening operation; their method was able to achieve 94.8% correct segmentation on a dataset of 1312 nodules, both attached and isolated. Other research on this topic includes a study by Shen et al. [8] which used a surface smoothing algorithm to segment nodules on the chest wall as well as a study by Kuhnigk et al. [9] which used a convex hull operation to perform segmentation. Similar to juxtapleural segmentation, lung nodule detection systems often require segmentation of the lung parenchyma from the chest wall while ensuring that juxtapleural nodules are included in the segmentation of the lung. Armato et al. proposed the use of a rolling ball filter to recover juxtapleural nodules removed from the lung segmentation [10], while Gurcan et al. used an indentation detection method based on a ratio of distances computed from tracing the contour of the lung [11]. Ko and Betke relied on a rapid change in curvature of the lung border to indicate structures, such as lung nodules or vessels, that were removed from the lung segmentation; these were recovered by the insertion of a border segment [12]. While these techniques show some preliminary success in segmenting juxtapleural nodules, many of these methods have difficulty in segmenting nodules in regions with moderate to high curvature or nodules whose shape is moderately complex, and all of these methods were evaluated on datasets with few juxtapleural nodules.

We propose a method to segment a nodule from the thoracic wall by fitting a polynomial function to the pleural surface. Surface-fitting methods have been used to solve problems in several different areas, including range [13, 14] and medical image data [15]. The approach explored in this paper relies on identifying pleural surface points and using these points to fit a polynomial surface function to the pleural surface. A statistically robust algorithm based on linear regression is used to identify relevant points and estimate surface parameters. This application is unique because it requires an accurate representation of a missing section of the pleural surface (the section due to the nodule) in the presence of an irregularity (nodule). Performance of the algorithm is measured by the number of “successful” segmentations, as assessed by visual observation, and the segmentation performance is compared to a previously published method on the same dataset of attached nodules.

## 2. Methods

The algorithm relies on several assumptions, the principal one being that the chest wall is a large surface with a curvature lower than the nodule surface. These assumptions are used in the development of a robust algorithm for juxtapleural nodule segmentation.

### 2.1. Juxtapleural Pulmonary Nodule Model

**Figure 2:**
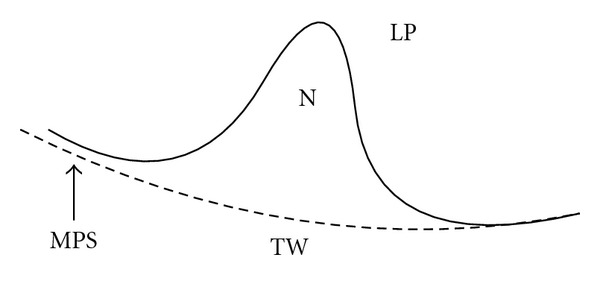
Juxtapleural nodule model, with the modeled pleural surface (MPS) separating the nodule (N) from the thoracic wall (TW). The lung parenchyma in the region indicated by LP.

 We model the juxtapleural nodule into several different regions, illustrated in Figure 2. These regions are the lung parenchyma (LP), the segmented thoracic wall (TW), the segmented nodule (N), and the modeled pleural surface (MPS). It is nearly impossible to determine whether the nodule has invaded the thoracic wall or is merely adjacent to it, so in our model, we consider only that portion of the nodule that is inside the lung volume to be part of our nodule region. In general, the pleural surface is a closed surface around the lung with many features. However, the scale of these features is much larger than that of the nodules. We expect the nodule to be the largest complete feature on the pleural surface in the region of interest. The pleural surface section within the region of interest should be smooth with a curvature much lower than that of the nodule surface; therefore, we can define a smooth function that is a global model for the pleural surface in the region of interest (MPS). In particular, the MPS accurately describes the location of the pleural surface inside the high voxel intensity region of the thoracic wall (TW) based on the cumulative pleural surface evidence provided by the visible surface. Therefore, we can consider the segmented nodule region to be defined by its boundary with the lung parenchyma on one side and the modeled pleural surface (MPS) on the other. The boundary is modeled as a cubic polynomial surface in the algorithm.

### 2.2. Algorithm

**Figure 3:**
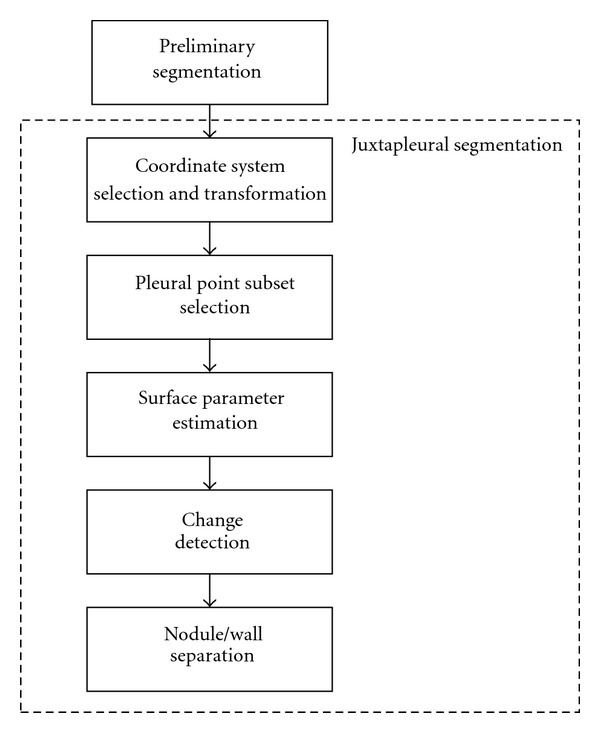
Flowchart overview of algorithm.

 The pleural surface is modeled as a 3D cubic polynomial function, and the goal of the surface-fitting algorithm is to determine the polynomial function that best fits the pleural surface. To accomplish this, the points belonging to the pleural surface are identified and used to estimate the parameters of the polynomial function. The algorithm is divided into the six stages shown in the flowchart in Figure 3.

The surface points in a region of interest are identified by first segmenting the nodule from the lung parenchyma and other soft tissue attached structures. After the preliminary segmentation of the nodule, the next task is to compute a coordinate transformation to assist in later stages of the algorithm that require computing the residuals from the estimated polynomial function. To ensure that the estimated polynomial function is computed from just the pleural surface points (excluding points belonging to the surface of the nodule), the next three steps encompass an iterative process that selects a subset of the surface points in the region, estimates a polynomial function from the subset of points, and determines if the change in residuals corresponds to the estimate of the polynomial surface that includes all the pleural surface points but none of the nodule surface points. Finally, in the last stage, the estimated surface function is used to segment the nodule from the pleural surface.

#### 2.2.1. Initial Setup

The surface-fitting algorithm is an extension of previous work on pulmonary nodule segmentation by Reeves et al. [5] that is designed to specifically address the task of separating nodules from the pleural surface. This algorithm relies on results of previous steps of the work by Reeves et al. which are summarized here.

The nodule segmentation system by Reeves et al. [5] can be divided into four main steps: (1) image preprocessing to select a region of interest based on a user-specified seed point within the nodule, (2) segmenting the nodule from the lung parenchyma by thresholding, (3) removing vessels, and (4) the elimination of the pleural surface using a clipping plane. This fourth step is replaced with the algorithm described in this paper.

In the image processing step, a small region of interest (ROI) sized more than twice the nodule diameter is extracted from entire CT scan centered at the seed point. From this ROI, an estimate of the nodule size and location is computed using an iterative template matching technique. This information is used to further reduce the size of the ROI. The small ROI is resampled into isotropic space (0.25 mm^3^ voxel size).

Next, the soft tissue in the resampled ROI is separated from the lung parenchyma by applying a threshold of −400 HU to the ROI. This results in a binary image with the lung parenchyma assigned a value of 0, and the soft tissue assigned a value of 1. To separate the nodule from other small, attached soft tissue structures, an iterative morphological filter is applied to the binary image which removes attached structures such as blood vessels but will not remove larger attached structures such as the pleural surface.

After these steps, we have the following information: (1) the volumetric region of interest resampled into isotropic space, (2) an approximate nodule radius, (3) an approximate nodule center, and (4) isotropic binary thresholded volume with the vessels removed. These are all used by the algorithm in subsequent steps.

#### 2.2.2. Coordinate System Selection and Transformation

The nodule is separated from the pleural surface by creating a boundary based on local shape information. The boundary is modeled as an explicit polynomial surface of the form

(1) z=F(x,y),

where one parameter (observable variable), *z*, can be described as a function of the other parameters (explanatory variables) *x* and *y*. The error measure of the estimated surface from the actual data points was defined as the discrepancy in the observable variable  *z*.

This explicit function requires finding the parametrization of the surface using techniques similar to those described by Quek et al. [16]. The first step of the method is the detection of the surface of the nodule and thoracic wall. From the binary image of the region of interest of the nodule obtained in Section 2.2.1, the surface is detected via an erosion operation with a spherical kernel 3 voxels (0.75 mm) in diameter followed by a logical XOR operation. This yields a binary image in which the voxels on the surface are 1 while all other voxels are 0. This binary image is used as a mask to select the regions of the grayscale image corresponding to the surface. The next step involves finding the surface function that has the least error compared to the actual surface.

**Figure 4:**
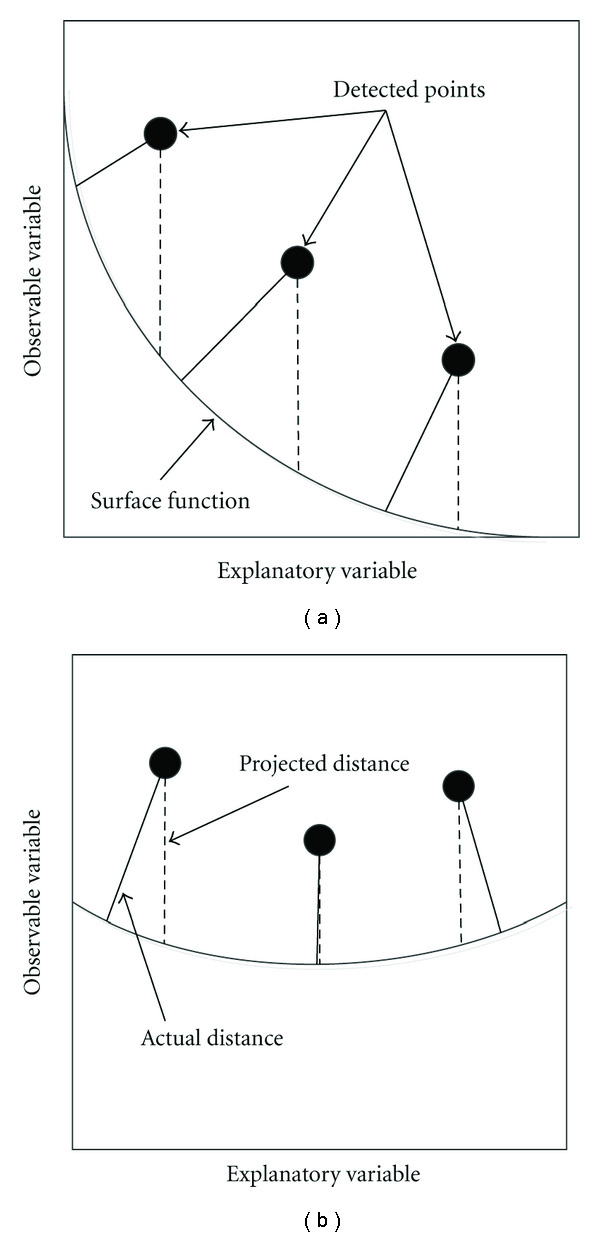
Estimating distances from a set of surface points to an estimated surface in (a) the original coordinate system, the error estimated by the observable variable (dashed lines) differs greatly from the actual error (solid lines), but if (b) the pleural surface is parallel to the explanatory variable plane, the error estimated by the observable variable provides a good approximation to the actual error.

**Figure 5:**
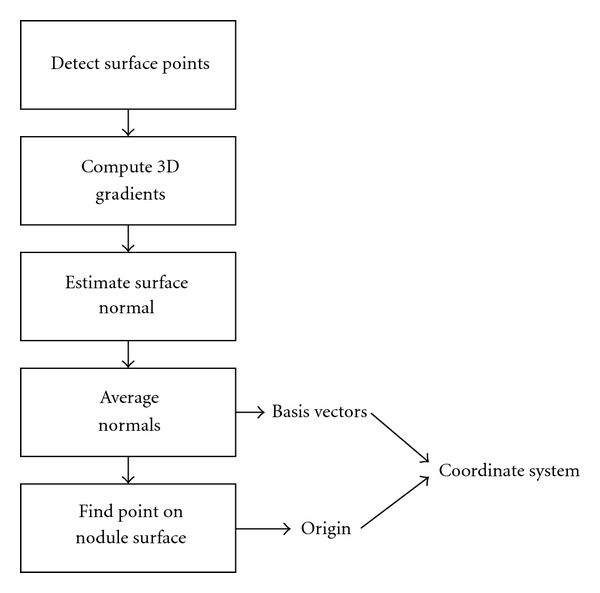
Overview of coordinate system selection.

 For an explicit surface function, we can improve the error computation by choosing a coordinate system that is parallel to the pleural surface. As shown in Figure 4(b), if the coordinate system is parallel to the pleural surface, estimating the error using the observable variable (*z*) exhibits much less error than in a different coordinate system, such as in Figure 4(a). An overview of the procedure for finding such a coordinate system is shown in Figure 5. First, the surface normals are estimated at all of the detected surface points using a 3D gradient operator. Second, the normal estimates are averaged to obtain an average normal estimate, $\overset{®}{N}$. The average normal is used as the *z*-direction basis vector with respect to (1), and the other two basis vectors, corresponding to *x* and *y* in (1), are selected to be orthogonal to the average normal and to each other.

**Figure 6:**
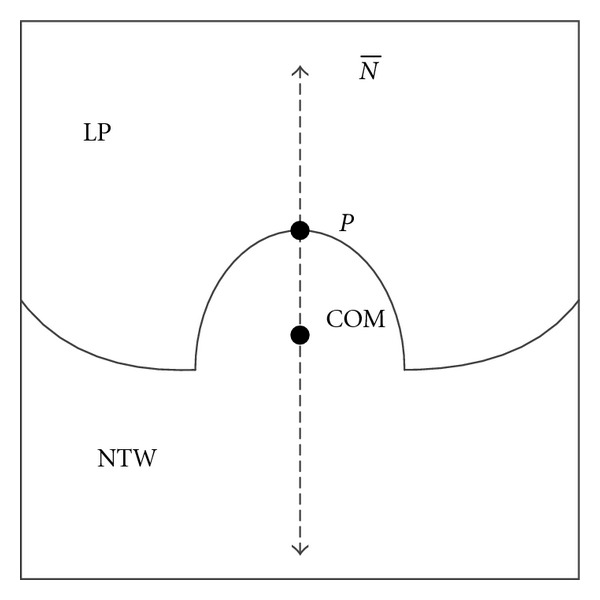
Procedure for finding nodule surface point. Beginning from the center of mass (COM), the algorithm searches for a surface point (*P*) in the direction of the average surface normal ($\overset{®}{N}$).

In addition to the basis vectors of the coordinate system, computing a coordinate transformation requires a point for the origin of the coordinate system. In this method, a reference point *P* on the nodule surface is used as the origin for the coordinate system. This reference point is identified by starting from a point within the nodule and searching in the direction of the average normal until the edge of the segmented region is detected, as shown in Figure 6. The point within the nodule is defined to be the nodule center of mass, which is found by computing the center of mass within the largest spherical region within the ROI centered at the approximate nodule center. The center of mass is used to ensure the point is consistent and because the estimate of the nodule center may have been affected by the presence of attached vessels, which have been removed prior to this step. Now all the surface points can be transformed into a coordinate system that has the nodule surface point at the origin and one of the basis vectors pointing in the direction of the normal. 

#### 2.2.3. Pleural Point Subset Selection: Modeling the Region of Interest

At this point, we have a coordinate system and points along the boundary of the thoracic wall/nodule region and the lung parenchyma. To prevent bias in the surface estimate, the surface points belonging to the nodule must be excluded. We use the following model as a basis for deciding which points belong to the nodule.

**Figure 7:**
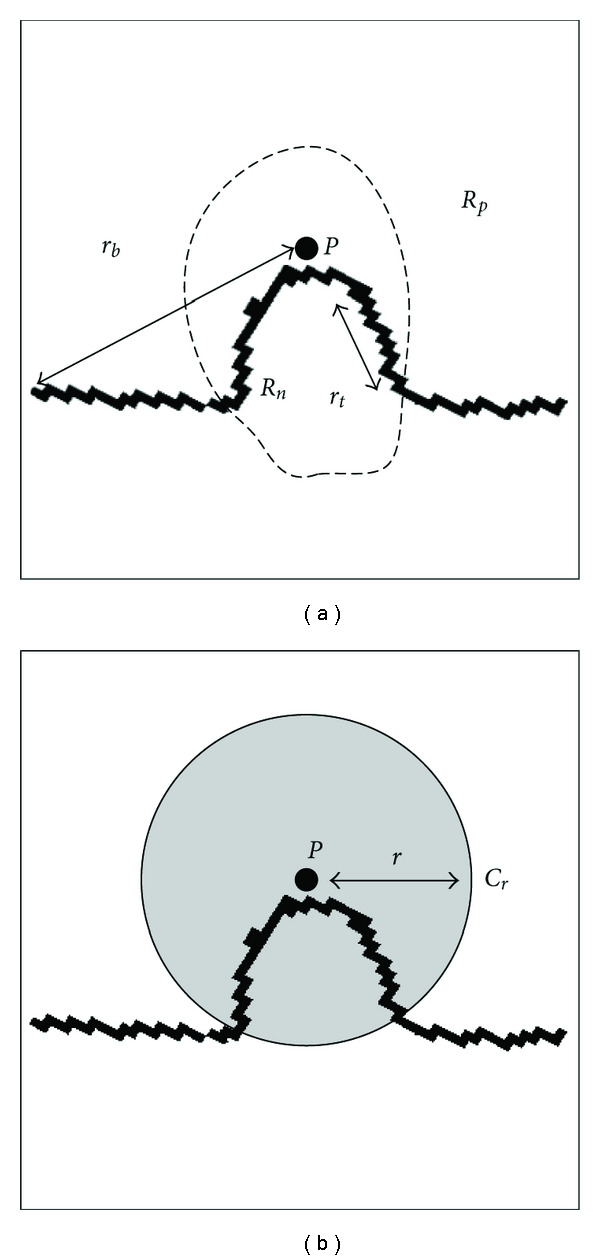
(a) Model of region of interest, with nodule subregion indicated by the dotted line and (b) region excluded for pleural point subset selection, indicated by gray-shaded circle (all surface points outside the circle, *C*_*r*_, are in the selected subset).

We consider the region of interest containing the nodule to consist of two sections: the pleural surface and the nodule surface. These sections can be separated by partitioning the region of interest into two subregions. One such partition is illustrated in Figure 7(a). In this illustration, *R*_*n*_, the region inside the dotted line, is the region containing the nodule, and *R*_*p*_, the region outside the dotted line, is the region containing only the pleural surface.

We define the following parameters that can be found without knowledge of the exact partition. A point that is known to be on the nodule surface is the reference point *P* found in the previous step. The minimum distance from *P* to a surface point adjacent to the edge of the region of interest is labeled *r*_*b*_. In addition, based on the region partition, we define *r*_*t*_ to be the maximum distance from *P* to another point in *R*_*n*_. In order to achieve a good segmentation, the nodule must be completely contained inside the region of interest. Although there are significant variations in nodule shapes, a generic partitioning region *C*_*r*_ can be defined as a region located outside of a sphere of radius *r* centered at the nodule surface point *P*, as shown by the region in white in Figure 7(b). Thus, *C*_*r*_ contains only pleural surface points when *r* > *r*_*t*_. In particular, *C*_*rb*_ is one set that can be easily generated and provides a good initial surface estimate, but with few surface points. A more robust solution can be found by increasing the number of points in the subset used for parameter estimation. The success of the algorithm depends on the strategy for picking the order of points to be added and finding a good stopping condition by detecting the presence of outliers.

Outliers or surface irregularities can be identified based on their inconsistency with the surface fit. A distance-based measure, such as mean squared error, can be used to determine the consistency of points with a surface model. To ensure that the mean squared error can be used to detect outliers, a surface model must be selected that can represent clean sections of the pleural surface but not ones containing the nodule. We make the following assumptions about the nodule surface: the nodule surface contains multiple inflection points, all the nodule surface points are on the lung side of the pleural surface, and the points that are farthest from the origin are adjacent to the pleural surface.

**Figure 8:**
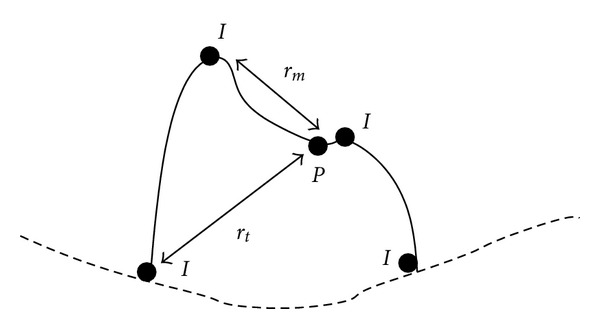
Model of a juxtapleural nodule with inflection points labeled as *I*, the distance from the origin (*P*) to the nodule periphery labeled *r*_*t*_, and the distance from the origin to the most distant peak labeled *r*_*m*_.

Inflection points are defined as points where the convexity of the surface changes. The presence of multiple inflection points differentiates a surface containing the nodule from the normal pleural surface. Several inflection points, labeled *I*, are seen in a two-dimensional nodule representation, shown in Figure 8, and even more can be found in the 3D image. The second condition ensures that the nodule is one connected region located exclusively inside the lung parenchyma. The third condition indicates that a nodule is a compact structure, where the distance from the origin to the nodule periphery,  *r*_*t*_, is larger than the distance to the farthest peak, labeled *r*_*m*_ in Figure 8. 

**Figure 9:**
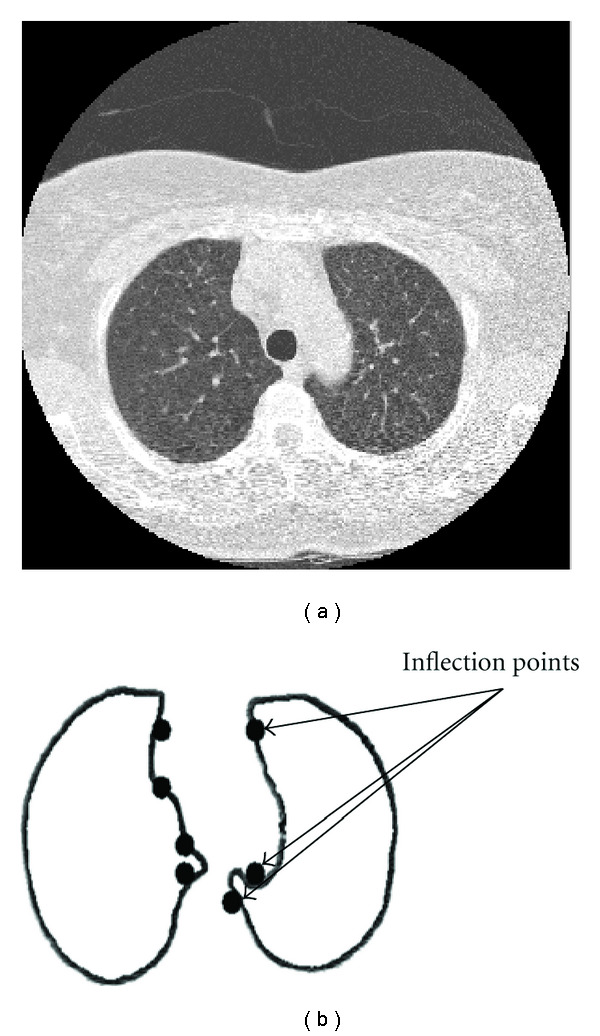
Pleural surface: (a) slice of a whole lung scan, (b) outline from a slice of a whole lung scan.

 In contrast to the nodule, the pleural surface has low curvature with few inflection points.  Figure 9 shows a typical slice of a whole lung scan and an outline of the pleural surface from this slice with inflection points marked by dots. In most cases, these inflection points are far apart from each other. In fact, no inflection points at all are apparent in the exterior lung region. On the other hand, the nodule attached to the pleural surface can be seen to have two inflection points in one slice with more present in the 3D image. Thus, in a small region of interest, the pleural surface should have very few inflection points compared to the nodule surface, which indicates that a family of second or third degree polynomial functions can be used to represent the pleural surface but not the nodule.

#### 2.2.4. Pleural Point Subset Selection and Parameter Estimation

**Figure 10:**
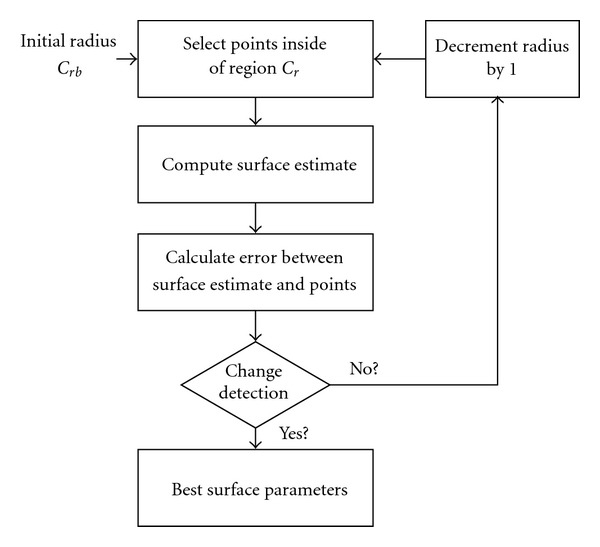
Overview of pleural point subset selection and surface parameter estimation.

 Once we have a method to select which surface points to include in our surface estimation algorithm, our next step is to find the optimal polynomial function that fits those points. An overview of the algorithm is shown in Figure 10.

The algorithm uses a forward search, starting with a small subset of points that is known to contain no or few outliers. The radius of the initial clean subset is initialized to a value of 10% higher than the known clean subset *C*_*rb*_, *r*_0_ = 1.1 · *r*_*b*_, to ensure that several diagnostic values for clean subsets are generated by the algorithm. At each step *i*, the subset radius is decreased in increments of *d*, in other words,  *r*_*i*_ = *r*_0_ − *i* · *d*. The algorithm uses a value of *d* = 1 voxel or 0.25 mm. Given the points in the subset, the parameters of the best-fitting polynomial function, *β*_*i*_ were calculated using least squares regression. The diagnostic value, *L*_*i*_, is defined as the average difference of the pleural surface points from the polynomial function added at the current step, as suggested by Atkinson and Riani [17],

(2) $$L_{i}=\sum_{\left((u,v,w)\in (C_{r_{i+1}}-C_{r_{i}}\right)}{\left(w-\beta_{i}^{T}X\right)}^{2},$$

where *X* = [*u*^*n*^, *u*^*n*−1^*v*, *u*^*n*−2^*v*^2^ ⋯ *uv*^*n*−1^, *v*^*n*^, *u*^*n*−1^ ⋯ *v*^*n*−1^ ⋯ *u*, *v*, 1]^*T*^. New points are added by reducing the radius of the clean subset. From this algorithm, we obtain a sequence of diagnostic values and corresponding polynomial function estimates.

**Figure 11:**
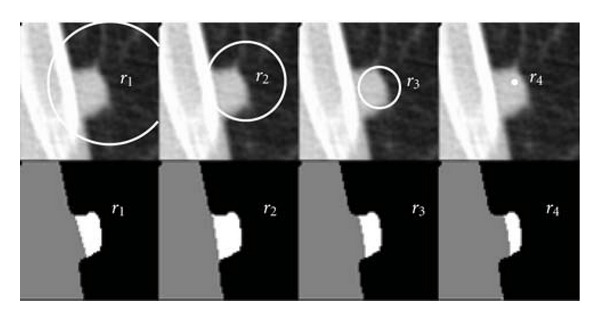
Example of forward search algorithm showing the central slice of a nodule, with the top row of images displaying several different radii of the region excluded from the subset of pleural surface points, and the bottom row of images displaying the resulting segmentations. Radius *r*_2_ contains the largest set of pleural surface points without including nodule surface points; including too many or too few points negatively affects the segmentation.

As the algorithm iterates between *r*_*b*_ and *r*_*t*_, we expect the diagnostic value to increase slowly, while the quality of the surface estimate improves. For subsets with radius less than *r*_*t*_, the diagnostic values will rise much faster, and the quality of the fit will deteriorate. Several iterations of the algorithm are shown in Figure 11, with the top row of images showing several subsets of points (points are those on the thoracic wall/nodule and lung parenchyma boundary outside of the area enclosed in the circle) and the bottom row showing the resulting segmentation images. Note that the best radius,  *r*_2_, includes as many points on the pleural surface as possible without including points on the nodule surface; subsets that include greater or fewer number of points have worse segmentation results. The appropriate subset can be found automatically by detecting a change in the behavior of the sequence of diagnostic values. 

To detect the change in behavior of the diagnostic sequence, we used a likelihood-based approach for constant level signals with noise described by Gustafsson [18], details of which are given below. Given a discrete-time signal *y*^*t*^ = *y*_1_ ⋯ *y*_*t*_, its likelihood based on the model

(3) $$y^{t}=\theta +e_{t},\;e_{t}\in N\left(0,R\right)$$

is denoted *p*(*y*^*t*^ | *θ*, *R*). The likelihood can be decomposed as a product of likelihoods of each point, due to independence

(4) $$p(y^{t}\;|\;\theta ,R)=\overset{t}{\underset{i=1}{\prod }}p\left(y_{i}\;|\;\theta ,R\right)={\left(2\pi R\right)}^{-t/2}e^{(-1/2\pi R)\sum_{i=1}^{t}{\left(y_{i}-\theta \right)}^{2}}.$$

For change detection, we are interested in the time where the signal changes, *k*. We can write parameters *θ* and *R* in terms of time by using the most likely parameters based on the sequence

(5) $$\begin{array}{c} {\hat{\theta }}^{ML}=\bar{y}=\frac{1}{t}\overset{t}{\underset{i=1}{\sum }}y_{i},\\ {\hat{R}}^{ML}=\bar{y^{2}}-{\bar{y}}^{2}=\frac{1}{t}\overset{t}{\underset{i=1}{\sum }}\left(y_{i}-{\hat{\theta }}^{ML}\right). \end{array}$$

*p*(*y*^*t*^ | *k*) represents the likelihood of measurement *y*^*t*^ given change time *k*. The most probable change time can be estimated by the maximum likelihood principle

(6) $$\hat{k}=\text{argma}{\text{x}}_{k}p\left(y^{t}\;|\;k\right).$$

For each *k*, the likelihood of the sequence can be decomposed into a product of two parts

(7) $$p\left(y^{t}\;|\;k\right)=p\left(y_{1:k}\;|\;k\right)\;p\left(y_{k+1:t}\;|\;k\right),$$

where *y*_*t*_1_:*t*_2__ = *y*_*t*_1__,…, *y*_*t*_2__.

Finally, the most likely sequence is found by minimizing the following expression:

(8) $$w_{k}=-2\log\left(p\left(y_{1:k}\;|\;k\right)\right)-2\log\left(p\left(y_{k+1:t}\;|\;k\right)\right),$$

where the likelihood of a subsequence can be calculated based on the estimated parameters

(9) $$-2\log\left(p\left(y^{k}\right)\right)=t\cdot \log\left(2\pi \right)+t+t\cdot \log\left({\hat{R}}^{ML}\right).$$

To find the change time for the sequence of diagnostic values, we formulate the problem using the framework just described. We treat the sequence of diagnostic values as a discrete time function and make the assumption that the function increases linearly, with a change in the slope of the function at the nodule radius, *r*_*t*_. We model the sequence in the interval  0 ⋯ *i*_*m*_, where *i*_*m*_ is the maximum element of the sequence, with the following expression:

(10) $$\begin{array}{c} L_{i}=\theta_{0}i+e_{0},\;i\leq i_{t}\\ L_{i}=\theta_{0}i_{t}+\theta_{1}\left(i-i_{t}\right)+e_{1},\;i>i_{t}, \end{array}$$

where the rate of increase before *i*_*t*_,  *θ*_0_, is much lower than the rate of increase after, *i*_*t*_, *θ*_1_, where nodule surface points are included in the subset. Error terms *e*_0_ and *e*_1_ are included in the expressions to represent the discrepancies between the model and actual residuals. When *i* ≤ *i*_*t*_, the error *e*_0_ is due to small features present on the pleural surface and is expected to be smaller than the error, *e*_1_, when *i* > *i*_*t*_ and the subset of surface points contain outliers that depend on the nodule shape.

To identify the time where the change in slope occurs, we can define a difference sequence *y*^*t*^,

(11) $$y^{t}⟵L_{2:i_{m}}-L_{1:(i_{m}-1)},$$

which results in the following model for the resulting sequence:

(12) $$\begin{array}{c} y_{i}=\theta_{0}+{e_{0}^{\prime }}_{}\;i\leq i_{t},\\ y_{i}=\theta_{1}+{e_{1}^{\prime }}_{}\;i>i_{t}. \end{array}$$

The change time can now be determined using the likelihood-based signal change detection method described by Gustafsson. The polynomial function associated with the change time will best fit the subset of the pleural surface points that excludes points on the nodule surface.

#### 2.2.5. Nodule Separation

**Figure 12:**
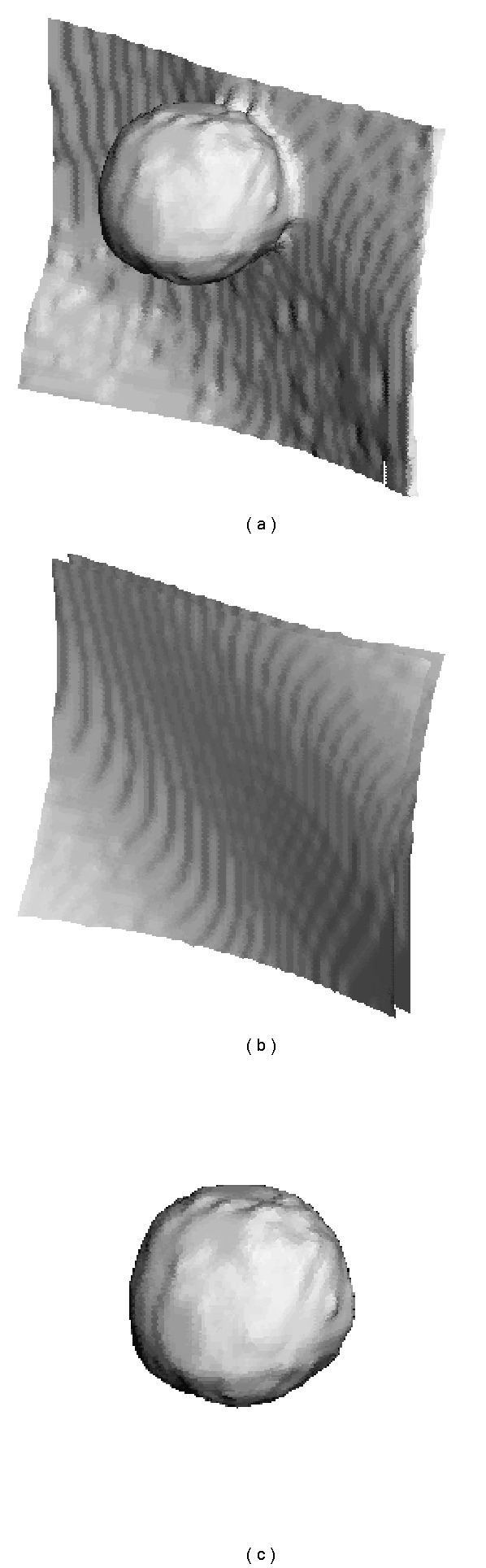
Thoracic wall removal. Light-shaded visualizations of (a) original region of interest, (b) estimated surface function, and (c) nodule after thoracic wall removal.

Once the surface parameters have been estimated, there is enough information to segment the nodule from the thoracic wall. We start with a binary image containing the thoracic wall and the nodule and eliminate voxels that are below the estimated pleural surface, where “below” is defined as the opposite direction of the average surface normal.  Figure 12(a) illustrates a 3D light-shaded model of a region of interest, with the surface estimate shown in Figure 12(b). Voxels below the surface are removed, leaving the nodule and some pixels due to small surface features and an imperfect representation of the surface. These can be removed by performing morphological opening followed by connected component analysis, and selecting the largest connected component. The results of segmentation are shown in Figure 12(c).

### 2.3. Materials

This study used a dataset of 150 solid attached nodules with one primary attachment from 114 patients selected from the Weill Cornell Medical Center database. The solid consistency of the nodules was confirmed by a radiologist; juxtapleural nodules were noted by a radiologist and confirmed by visual inspection. Of the 150 nodules, six nodules were on whole-lung scans while the remainder were on targeted scans. All nodules were imaged on thin-slice scans, with 129 nodules on 1.00 mm scans and 21 nodules on 1.25 mm scans. All of the scans were acquired using the scanners and parameters shown in Table 1. The nodules ranged in size from 1.5 mm to 22.6 mm, as determined by a semiautomated volume measurement method, with a mean size of 5.2 mm. 

### 2.4. Experiment

**Figure 13:**
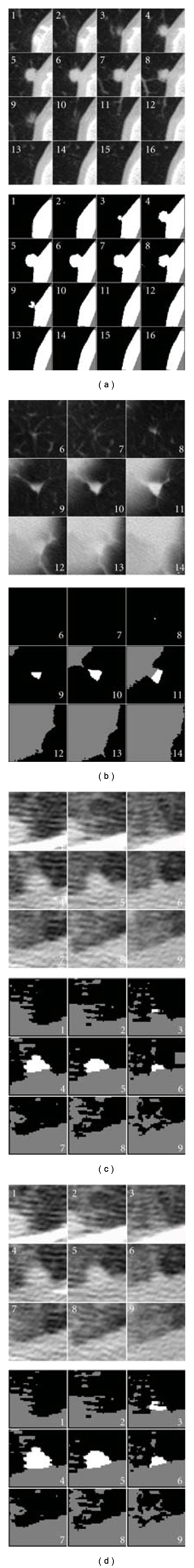
Examples of nodule segmentation ratings, (a) the segmentation includes extensive nonnodule regions, (b) the segmentation does not include the entire nodule, (c) the segmentation stops slightly short of the wall, and (d) the segmentation includes all of the nodule but none of the wall. Segmentations with a rating of 3 or 4 were considered acceptable. The top image shows the grayscale region of interest, while the bottom image shows the segmentation, with the nodule indicated in white and high-intensity nonnodule structures indicated by gray. Images were all windowed to enhance visibility and are not to scale.

The “true” segmentation of a juxtapleural nodule is difficult to accurately determine, even for radiologists. Studies have shown that there is high interobserver variability in nodule measurements [19, 20]. Thus, instead of directly comparing the overlap of the segmented regions, the segmentations for each nodule and method were visually inspected and ranked on a scale of 1 to 4, with 1 representing completely unacceptable segmentation and 4 representing very good segmentation for the purpose of volumetric measurement. Segmentations with a rating of 3 or 4 were considered to be acceptable for volumetric evaluation. Three raters (A. C. Jirapatnakul, A. P. Reeves, and D. F. Yankelevitz) reviewed the segmentations and arrived at a consensus. D. F. Yankelevitz is a board-certified radiologist. Examples of segmentations and their associated ratings are shown in Figure 13. The raters were presented with all slices of the region of interest around the nodule with the segmented regions overlaid on top of the original CT image region in a translucent color. To prevent bias, the raters were not aware of which segmentation method was being presented nor the order of presentation of the methods. All nodules were read in a single session. For the intersection of the set of nodules that were judged to have acceptable segmentations by both methods, the volumes were compared to determine if there was a significant difference between the methods. The initial seed point, region of interest, and other parameters were consistent across methods, with the initial seed point manually specified.

## 3. Results

The new surface-fitting method was compared to the latest published method from Reeves et al. [5]. Of the 150 nodules in the database, the surface-fitting algorithm acceptably segmented 147 nodules (98.0%), while the plane-cutting method by Reeves et al. acceptably segmented 122 nodules (81.3%). These results are summarized in Table 2. 

**Figure 14:**
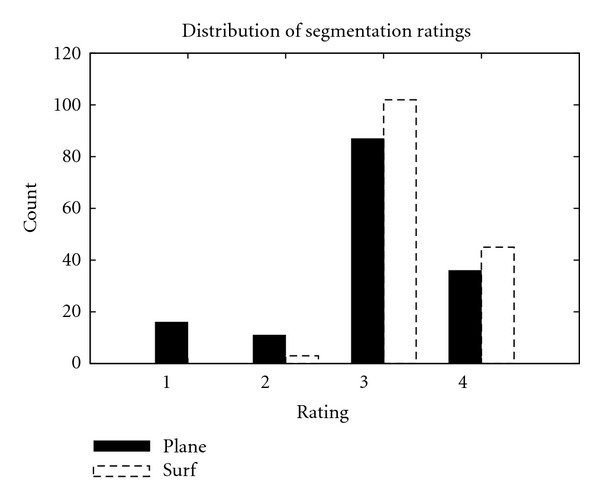
Distribution of ratings for segmentations by the surface-fitting and plane-cutting methods.

The average rating of the surface-fitting algorithm was 3.28 over all the nodules, while the average rating for the algorithm by Reeves et al. was 2.95, with the distributions shown in Figure 14. The volumes measured by both methods were compared using a paired *t*-test, which indicated statistically significant differences between the methods (*P* < 0.01). This did not change if we limited the analysis to the 122 nodules successfully segmented by both methods (rating of 3 or 4) (*P* < 0.01). The median volume difference of these 122 nodules was 5.7%, with only 17 nodules differing by more than 20%. There was no clear relationship between the size of the nodule and the success of the algorithm. 

The runtimes of both the methods were measured on a dual-processor Intel Xeon 3.0 GHz computer. Both methods were implemented in unoptimized research software. For most nodules, the runtimes of both methods were only a few seconds. The surface-fitting algorithm was slower than the plane-cutting method, with a range of runtimes of approximately 200 ms to 14 seconds. The plane-cutting method had runtimes which ranged from approximately 100 ms to 10 seconds. The runtimes of both methods were higher for larger nodules.

## 4. Discussion

A new algorithm for juxtapleural nodule segmentation was developed which combined robust surface estimation methods with knowledge of the characteristics of juxtapleural nodules to improve upon previous segmentation algorithms without requiring any additional user intervention. Although the majority of the nodules in this study were on targeted CT scans, the algorithm should work effectively on whole-lung CT scans as well.

**Figure 15:**
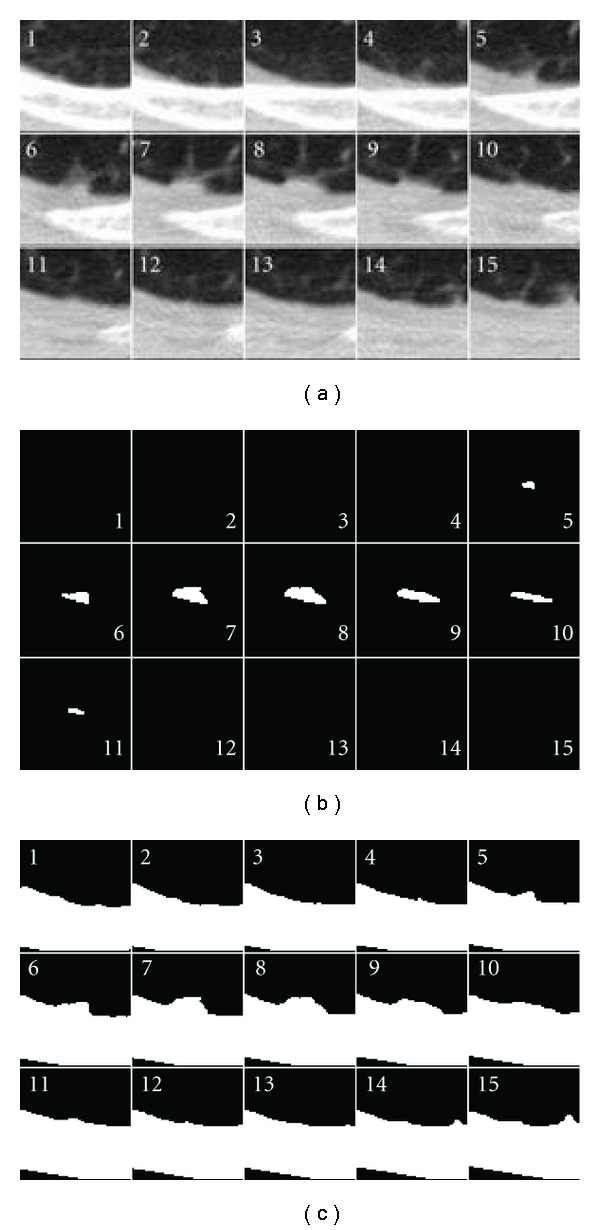
Example of a nodule where the segmentation performed by the surface-fitting algorithm is better than the segmentation performed by the plane-cutting method, with (a) the region of interest containing the nodule, (b) the nodule segmentation via the surface-fitting algorithm, and (c) the segmentation by the plane-cutting method. In (b) and (c), areas of white indicate nodule.

Unlike previous studies which reported their segmentation results on both isolated and attached nodules, the dataset used in this study consisted of only the more challenging juxtapleural nodules. On this set of juxtapleural nodules, this new algorithm performed better than a previously published method [5], which used a plane to represent the pleural surface, due to its ability to better model curved sections of the pleural surface. In our testing, the surface-fitting algorithm successfully segmented 98.0% of the attached nodules in our database, as compared to the previous algorithm using an iterative plane-cutting approach which only succeeded on 81.3% of nodules in the database. Most of the improvement came from nodules located on highly curved regions of the pleural surface where a plane would not be able to accurately represent the pleural surface. An example of a case where the surface-fitting algorithm is markedly better than the plane-cutting algorithm is shown in Figure 15. The surface-fitting algorithm is able to accurately separate the nodule from the wall, while the plane-cutting method segments a large portion of the wall. This is due to the fact that the pleural surface in this region is curved while the nodule is small, so the plane immediately includes portions of the wall. 

**Figure 16:**
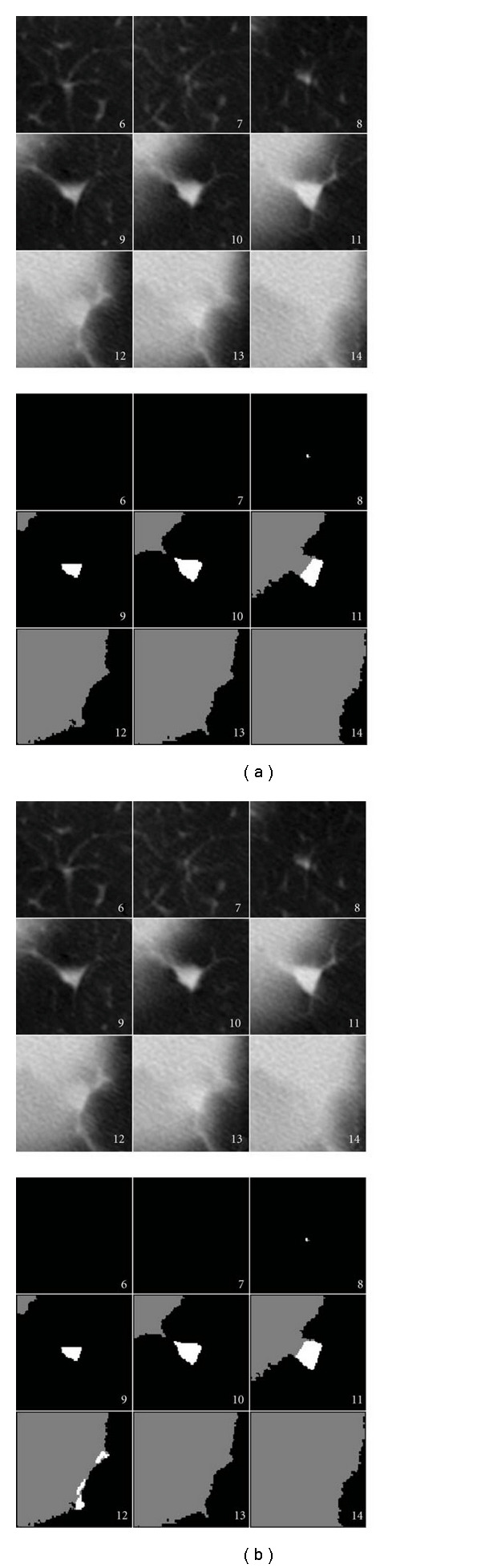
Several slices of the region of interest containing a nodule that the (a) surface-fitting and (b) plane-cutting methods failed to properly segment. The nodule was located near the diaphragm; notice that there was a large change in the position of the wall in frames 9 to 11. The voxels included in the segmentation are indicated in white, while high-intensity nonnodule voxels are shown in gray.

**Figure 17:**
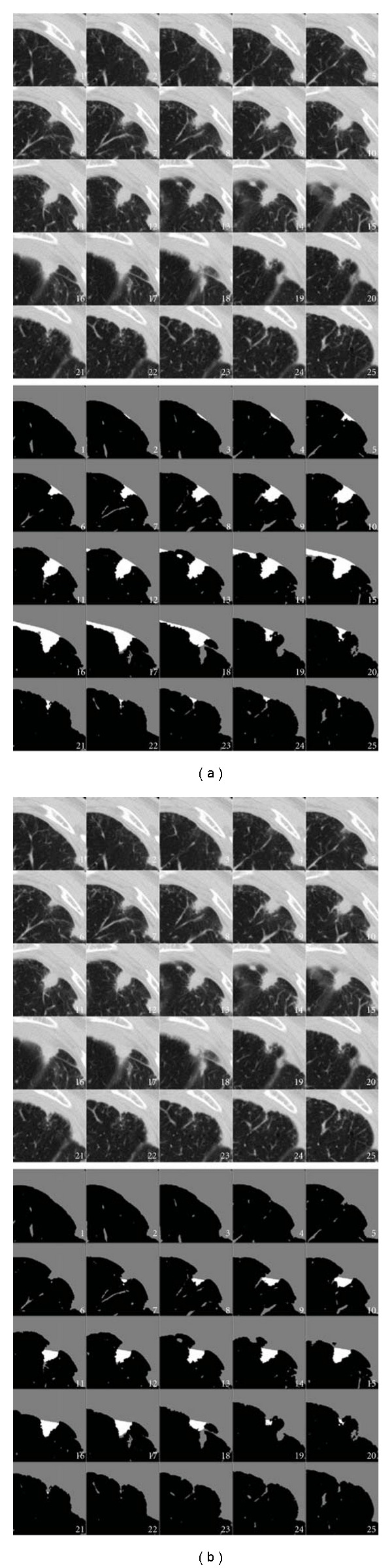
Example of a nodule for which both algorithms segmented a portion of the wall due to respiratory motion in frames 13–18. The voxels included in the segmentation are labeled in white for the (a) surface-fitting method and (b) plane-cutting method.

**Figure 18:**
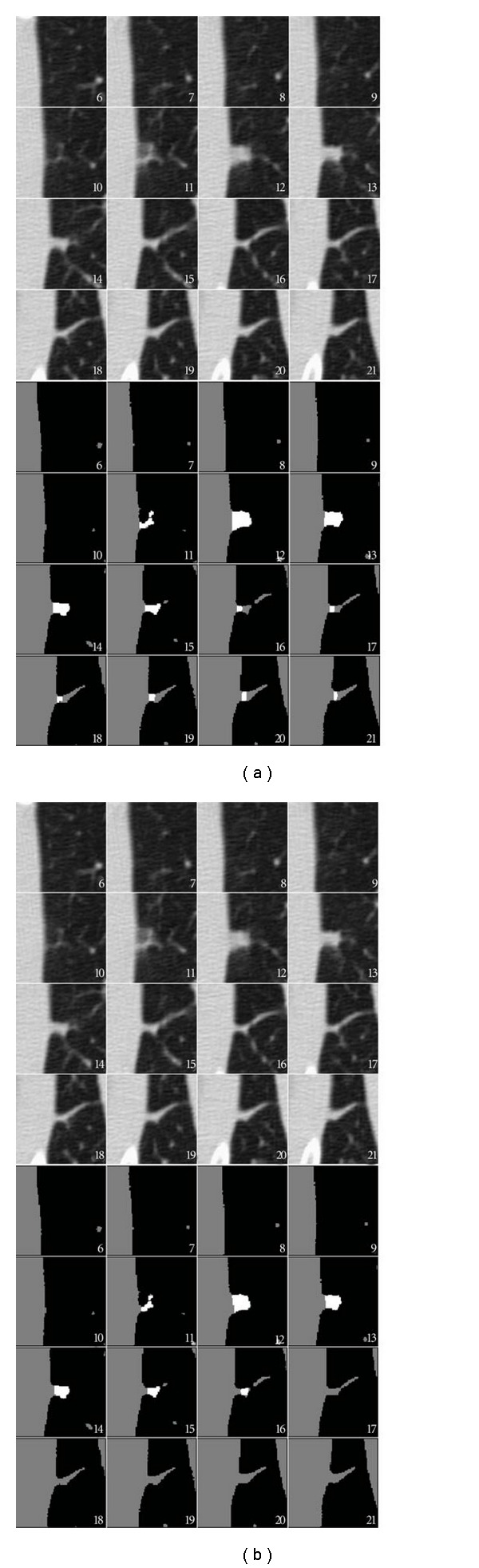
Example of oversegmentation by both algorithms. (a) An attached structure is included in the segmentation by the surface-fitting method as well as the (b) plane-cutting method, though not to as great an extent.

Although there was an improvement in the success rate of nodule segmentation with the surface-fitting algorithm, there were still several cases where both algorithms failed. Many of these failures were due to either respiratory motion, or an apparent shift of the nodule attachment point along the pleural surface. In one case, the nodule was located near the diaphragm. Several CT scan slices of the region of interest for this nodule are shown in Figure 16. Due to the high curvature of the diaphragm, the juxtapleural surface appears to move by a large amount between frames 9 and 11. The surface-fitting approach does not fully segment the nodule, due to not being able to accommodate the rapid change in position of the diaphragm. The plane-cutting method was able to get more of the nodule, but it included portions of wall. In another case, shown in Figure 17, respiratory motion caused the movement of the pleural surface in several slices; on these slices (frames 13–18), the surface-fitting algorithm incorrectly segmented portions of the wall. Aside from those frames, the segmentation is acceptable. While both algorithms were affected by this motion, the surface-fitting algorithm failed because it was not able to compensate for the shift, resulting in the segmentation of a large part of the pleural surface, whereas the plane-cutting algorithm was not able to get very close to the pleural surface, but was able to avoid segmenting the pleural surface. In the second case, the nodule was located near the diaphragm with a vessel-like attachment. The surface-fitting algorithm included portions of the attachment in the segmentation, whereas the plane-cutting algorithm did not, as shown in Figure 18. 

The runtime of the surface-fitting method was slightly longer than the runtime required for the plane-cutting method. Much of the runtime occurred in the iterative process of selecting a set of pleural surface points and using these points to estimate the parameters of a polynomial function. The runtime could be dramatically reduced by additional program optimizations, possibly by a factor of 10 or more for the larger nodules.

In this study, the segmentation results were subjectively evaluated by a three raters. Having a radiologist manually contour, every nodule on each slice is time-consuming, and though the Lung Image Database Consortium (LIDC) [21] provides contours for nodules in the database greater than 3 mm, previous studies have shown a large interobserver variation between radiologists [20, 22]. Additionally, the dataset used for evaluation in this study contained many more juxtapleural nodules than the LIDC database. Given these considerations, consensus ratings from visual inspection was used for this study.

## 5. Conclusion

We have presented a robust, surface estimation approach to accurately segment solid juxtapleural nodules. In contrast to previous approaches using morphological filtering, plane-cutting, or convex hull operations, this approach fits a polynomial function to a robust set of pleural surface points. We evaluated the performance of this algorithm on a database of 150 solid juxtapleural nodules and compared its performance to a previously published method using an iterative plane-cutting algorithm. Our method performs much better than the plane-cutting approach, correctly segmenting 98% of the nodules compared to 81% with the previous method. The surface estimation approach especially excels with nodules attached to pleural surfaces with high curvature. However, the algorithm is still affected by image problems such as respiratory motion, but this will become less of a problem with improvements in CT scanner technology. This approach improves the success rate of juxtapleural nodule segmentation and will allow for more accurate volumetric measurement of juxtapleural pulmonary nodules.

##  Conflict of Interests

D. Yankelevitz is a named inventor on a number of patents and patent applications relating to the evaluation of diseases of the chest including measurement of nodules. Some of these, which are owned by Cornell Research Foundation (CRF), are nonexclusively licensed to General Electric. As an inventor of these patents, D. Yankelevitz is entitled to a share of any compensation which CRF may receive from its commercialization of these patents. C. I. Henschke is a named inventor on a number of patents and patent applications relating to the evaluation of disease of the chest including the measurement of nodules. Some of these patents, which are owned by the Cornell Research Foundation (CRF), are nonexclusively licensed to GE Healthcare. As an inventor, C. I. Henschke is entitled to a share of any compensation that CRF may receive from the commercialization of these patents but has renounced any compensation since April 2009. A. P. Reeves is a coinventor on patents and pending patents owned by Cornell Research Foundation, which are nonexclusively licensed to GE and related to technology involving computer-aided diagnostic methods, including measurement of pulmonary nodules in CT images.

## Figures and Tables

**Table 1:** Image acquisition parameters.

Parameter	Value
Scanner	GE HiSpeed CT/i, LightSpeed Ultra, LightSpeed QX/i
Current	40–340 mA
Voltage	120, 140 kVp

**Table 2:** Nodule segmentation results comparing the surface-fitting method described in this paper with a previously published method using a plane-cutting approach.

Method	Nodules segmented	Percent segmented
Surface fitting	147	98.0%
Reeves et al. [5]	122	81.3%

Total number of nodules	150	
